# Association between Anxiety Levels and Weight Change in the Multiethnic Study of Atherosclerosis

**DOI:** 10.1155/2014/894627

**Published:** 2014-10-08

**Authors:** Katherine Rieke, Ramon Durazo-Arvizu, Kiang Liu, Erin D. Michos, Amy Luke, Holly Kramer

**Affiliations:** ^1^Department of Public Health Sciences, Loyola University Chicago, 2160 South First Avenue, Maywood, IL 60153, USA; ^2^Department of Epidemiology, University of Nebraska Medical Center, 984395 Nebraska Medical Center, Omaha, NE 68198-4395, USA; ^3^Department of Preventive Medicine, Feinberg School of Medicine, Northwestern University, 680 North Lake Shore Drive, Suite 1400, Chicago, IL 60611-4402, USA; ^4^Division of Cardiology, Johns Hopkins University School of Medicine, 600 N. Wolfe Street, Baltimore, MD 21287, USA; ^5^Department of Medicine, Division of Nephrology and Hypertension, Loyola University Chicago, 2160 South First Avenue, Maywood, IL 60153, USA

## Abstract

*Objective*. To examine the association between anxiety and weight change in a multiethnic cohort followed for approximately 10 years. *Methods*. The study population consisted of participants of the multiethnic study of atherosclerosis who met specified inclusion criteria (*n* = 5,799). Weight was measured at baseline and four subsequent follow-up exams. Anxiety was analyzed as sex-specific anxiety quartiles (QANX). The relationship between anxiety level and weight change was examined using a mixed-effect model with weight as the dependent variable, anxiety and time as the independent variables, and adjusted for covariates. *Results*. Average annual weight change (range) was −0.17 kg (−6.04 to 4.38 kg) for QANX 1 (lowest anxiety), −0.16 kg (−10.71 to 4.45 kg) for QANX 2, −0.15 kg (−8.69 to 6.39 kg) for QANX 3, and −0.20 kg (−7.12 to 3.95 kg) for QANX 4 (highest anxiety). No significant association was noted between QANX and weight change. However, the highest QANX was associated with a −2.48 kg (95% CI = −3.65, −1.31) lower baseline weight compared to the lowest QANX after adjustment for all covariates. *Conclusions*. Among adults, age 45–84, higher levels of anxiety, defined by the STPI trait anxiety scale, are associated with lower average baseline weight but not with weight change.

## 1. Introduction

The concept of emotional eating theorizes that people use food as a form of coping behavior when faced with negative feelings. While several studies have shown that psychological factors may be associated with disinhibited or disordered eating patterns, poorer dietary choices, and obesity [1], some scientific evidence supports the notion that physiological effects of stress and anxiety should suppress hunger in normal populations [2]. Overall, however, studies focusing on the impact of psychological factors on weight change have been inconclusive because investigators have largely focused on populations that are obese, dieting, or have disordered eating [3]. Additionally, most studies have been cross-sectional or focused on eating behaviors, and therefore, have not examined the long-term association between psychological factors and weight status.

Given the potential links between psychological factors, eating behaviors, and obesity, we examined the association between anxiety and weight change in a multiethnic cohort followed for approximately 10 years. We hypothesized that higher levels of anxiety are associated with reduced weight gain over time.

## 2. Methods

### 2.1. Study Participants

The multiethnic study of atherosclerosis (MESA) is a prospective cohort study of 6814 men and women aged 45 to 84 years without clinical cardiovascular disease (CVD), recruited from six U.S. communities (Baltimore, MD; Chicago, IL; Forsyth County, NC; Los Angeles County, CA; northern Manhattan, NY; and St. Paul, MN). MESA is designed to determine the characteristics of subclinical CVD and its progression. Information on the sampling frame and study design has been previously reported [4]. Participants who self-reported their race/ethnicity group as Caucasian or white, African-American or black, Chinese, or Spanish/Hispanic/Latino were asked to participate and were enrolled between July 2000 and August 2002. Four subsequent exams were completed with each exam occurring approximately every 1.5 years. Institutional review board approval was obtained at all MESA sites, and informed consent was obtained from all participants. Exclusion criteria for the MESA study included individuals with a history of CVD, individuals with impaired cognition, pregnant women, individuals weighing over 300 pounds at baseline, and anyone speaking languages other than English, Spanish, Mandarin, or Cantonese.

**Figure 1:**
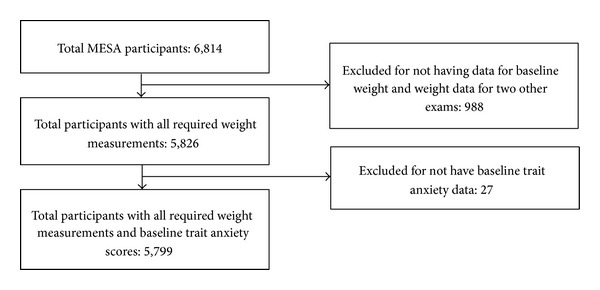
Flow chart of participant exclusion.

For this analysis, we excluded MESA participants without at least three consecutive weight measurements (*n* = 988). We also excluded participants with missing data on baseline trait anxiety scores from the Spielberger State-Trait Personality Inventory (*n* = 27) leaving a total of 5,799 MESA participants included in the analysis.  Figure 1 shows the selection of the MESA participants included in the analysis.

### 2.2. Anxiety

All MESA participants completed the Trait Anxiety Scale from the Spielberger State-Trait Personality Inventory (STPI), which measures long term, pervasive anxiety traits. The STPI trait scale consists of 10 questions about the general tendency to experience anxiety across time and situations. Each question is scored on a 1–4 point scale. Total scores range from 10 to 40 with higher scores indicating higher levels of anxiety [5].

### 2.3. Weight and Covariates

Height and weight were measured using a balanced scale and stadiometer, with participants wearing light clothing and no shoes [6]. All MESA subjects completed self-administered questionnaires and were interviewed by trained research staff in order to collect information pertaining to demographic characteristics, medical history, alcohol and tobacco use. Participants were asked to bring their medications to the exam, which were then recorded by MESA interviewers. These self-administered questionnaires were available in English, Spanish, Mandarin, or Cantonese.

Information on socioeconomic factors including highest degree or level of school completed, total household income, financial strain, use of medical services, and health insurance was collected from the MESA participants using questionnaires. The total household income included money from jobs, net income from business, farm or rent, pensions, dividends, welfare, social security payments, and any other incomes received by the participant and all household members living with the participant. Participants were instructed to choose one of thirteen income categories that best represented the total family income for the past 12 months. To estimate mean income, the midpoint of the range for each category was used. For the highest category income ($100,000 and higher), $120,000 was assigned. All covariate data were taken from the baseline exam and, therefore, were not dependent on time.

## 3. Statistical Methods

We first examined a locally weighted scatterplot smoother (LOWESS) graph of anxiety scores versus weight change as a continuous variable, which demonstrated a nonlinear association between anxiety and weight change. Therefore, anxiety scores were analyzed as sex-specific trait anxiety quartiles (QANX; 1 = first, 2 = second, 3 = third, and 4 = fourth quartile) based on the STPI scoring manual, as these quartile scores differ by sex. Baseline characteristics of the MESA participants were then compared across categories of QANX. Continuous variables were compared using analysis of variance and categorical variables were compared using the chi-square test. The level for statistical significance was set at *P* < 0.017 (0.05/3) to account for multiple comparisons (QANX 1 versus 2, 3, and 4).

We used multilevel mixed-effect models [7] with weight as the dependent variable and anxiety scores as the independent variable. Time entered the model as a random effect, whereas all other variables were entered as fixed-effects. This mixed-effects model used both participant level measures (Level 1) and weight determinations over time (Level 2), with weight determinations over time nested within the participant level measures. Let QFST_*i*_, QSND_*i*_, QTRD_*i*_, and QFTH_*i*_ dummy variables indicate whether the *i*th participant belongs to the first, second, third, or fourth quartile of trait anxiety score, respectively. The weight of the *i*th individual measured at the* j*th exam is denoted by *W*_*ij*_, and the time elapsed between baseline and the* j*th exam is represented by *T*_*ij*_, *i* = 1,…, *N*; *j* = 1,…, 6, with *T*_0*i*_ ≡ 0 for all *i*

(1) Level  1: Wij=β0i+β1i×Tij+εij Level  2:   β0i=γ0+γ1QSNDi+γ2QTRDi+γ3QFTHi+ξ0iβ1i=α0+α1QSNDi+α2QTRDi+α3QFTHi+ξ1i.

The *ε*_*ij*_ term was the random error associated with the* j*th exam weight determination of the *i*th participant and had a constant variance of *σ* ^2^. The baseline weight (*β*_0*i*_) and the rate of weight change over time for the *i*th participant (*β*_1*i*_) depended on the trait anxiety quartile (QSND, QTRD, and QFTH), and were also assumed to vary randomly across persons with variances of *δ*_0_^2^ and *δ*_1_^2^, respectively. All error terms were assumed to have a normal distribution with a mean of zero.

Since anxiety score was examined as a categorical variable, where the first quartile is the reference, *γ*_0_ represented the estimated mean weight at baseline among participants in the first quartile, *α*_0_ represented the estimated average weight change per year in the first quartile, *γ*_1_, *γ*_2_, and *γ*_3_ represented the estimated baseline average weight difference between the second, third, fourth quartile and the first quartile of trait anxiety, respectively. The rate of weight change per year in the second, third, and fourth quartiles were estimated by *α*_1_, *α*_2_, and *α*_3_, respectively. The model above can be rewritten as

(2) Wij=γ0+γ1QSNDi+γ2QTRDi+γ3QFTHi +α0Tij+α1QSNDi×Tij+α2QTRDi×Tij +α3QFTHi×Tij+ξ1iTij+ξ0i+εij         i=1,…,N; j=1,…,6.

This unadjusted model is termed Model 1, whereas the adjusted model (Model 2) included age race/ethnicity and sex, and the third model (Model 3) added education level, income level, marital status, and MESA site to Model 2.

## 4. Results

Overall, 52.5% of the participants were female and mean age at baseline was 61.7 years. Mean baseline weight was 78.87 kg (range 32.51 to 146.10 kg). The total average weight change was −1.28 kg for men and −1.08 kg for women over the 10 year period. Baseline weight by race/ethnicity was 79.60 kg (range 39.04 to 136.65 kg) for Whites, 63.34 kg (range 38.36 to 112.59 kg) for Chinese Americans, 85.66 kg (range 39.50 to 142.74 kg) for African Americans, and 77.62 (range 32.51 to 146.10 kg) for Hispanic participants.  Table 1 shows the ranges of STPI scores by sex-specific quartiles.

Table 2 shows baseline characteristics for participants by trait anxiety score quartile, with mean and standard deviation values reported for continuous variables and percentages reported for categorical variables. Baseline weight and age were lowest in the highest quartile (highest anxiety) but differences across anxiety score categories for baseline weight and age were not statistically significant. The percentage of white and Chinese participants was highest in the fourth quartile and lowest in the first quartile, while the percentage of black participants was lowest in the first quartile and highest in the fourth quartile. Income also differed significantly by anxiety quartile, with the highest percentage of those with incomes less than $20,000 being in the fourth quartile. Marital status differed significantly by anxiety quartile with those in fourth quartile of anxiety score having the highest percentages of individuals who reported being separated, or never married and those in the lowest quartile having the lowest percentage of those who reported being widowed. The percentage of individuals with no schooling was highest in the fourth anxiety quartile, and the percentage of those reporting having some college-no degree and above being lowest in the fourth anxiety quartile.

**Figure 2:**
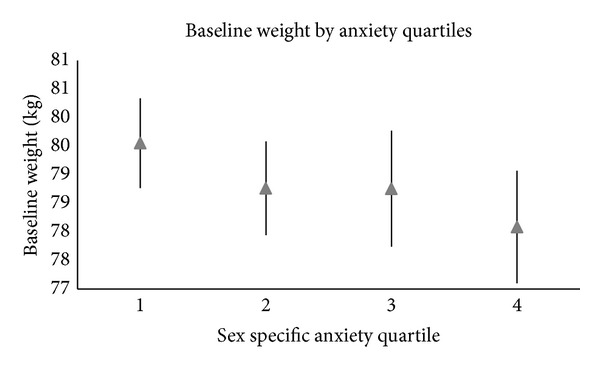
Graph of the mean and related confidence intervals of the baseline weight (kg) by sex-specific anxiety quartiles.

**Figure 3:**
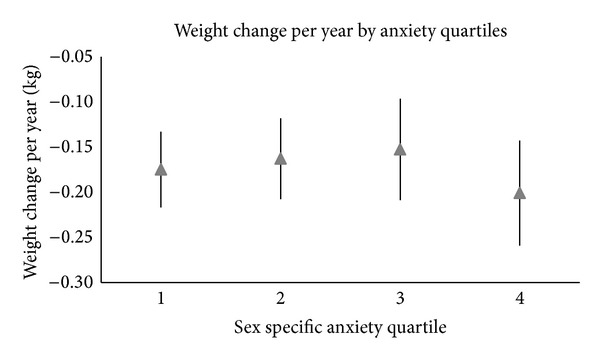
Graph of the mean and related confidence intervals of the change in weight per year (kg) by sex-specific anxiety quartiles.

Figure 2 shows the mean baseline weight (kg) and 95% confidence intervals by trait anxiety score quartile while Figure 3 shows the mean weight change (kg) per year and 95% confidence intervals by anxiety quartile. Mean weight change was −0.17 in the lowest anxiety quartile to −0.20 in the highest.

In the mixed effects model, a significant association was noted between trait anxiety score and baseline weight. After adjustment for all covariates, the highest anxiety quartile was associated with a 2.48 kg lower average baseline weight (95% CI = −3.65, −1.31) compared to the lowest (Table 3). The second trait anxiety quartile was associated with 1.22 kg (95% CI = −2.26, −0.18) lower baseline weight compared to the lowest while the third quartile was associated with 1.61 kg (95% CI = −2.81, −0.41) lower baseline weight compared to the first.

Table 4 shows the beta-coefficients and standard errors for the association between anxiety score category and weight change. Results from all models (Models 1–3) showed no significant association between trait anxiety score and weight change over time (represented by *α*_1_, *α*_2_, and *α*_2_ in the models above). For instance, Model 3 estimated regression coefficients for interaction of anxiety score category on weight change were −0.02 (*P* > 0.05) or less for the second, third and fourth quartile, respectively. Because of the significant association between anxiety and baseline weight, the interaction of anxiety score with race, age, and gender were examined individually. No significant interactions were found.

## 5. Discussion

After accounting for the within-person variability of weight change, we found no significant association between sex specific baseline trait anxiety score quartiles and weight change over time in this multiethnic cohort of adults without established clinical CVD at baseline. However, our results showed a significant association between sex specific baseline anxiety score quartiles and baseline weight. It is possible that the null association we noted between sex specific anxiety score quartiles and weight change yet a significant association with baseline weight may be due to the older age of the MESA cohort (45–84 years at baseline, mean age 62). Other studies which noted a significant association between anxiety scores and weight change focused on a much younger population. One previous study found that both gender and age of onset of psychological symptoms may play a significant role in the trajectory of anxiety's influence on weight over time [8]. Since adolescence is a time where weight increases rapidly and eating behaviors may be most impacted by environmental factors [9], anxiety may have a greater impact on weight gain during this time compared to later in life. Additionally, there is a general decline in weight that occurs in older adults due to in part decreased food intake [10], and in our study the average weight change was negative. This decrease in food intake, related partially to a reduction in hedonic qualities of food and a slower rate of gastric emptying in aging populations [10], may lead to fewer differences in weight change among older populations.

The strengths of the study include the use of a multiethnic cohort and standardized measurements of weight. The longitudinal study design allowed the assessment of temporality which may be precluded with a cross sectional study design. Additionally, the study population was not focused on individuals with known disordered eating or obese individuals, and therefore may be more generalizable to nondisordered eating populations. Weaknesses of the study include the use of a single point measure of anxiety. While trait anxiety is thought to be relatively stable over time, there may have been changes in participants' trait anxiety scores over the 10 year period. Additionally, the data do not provide information on clinical or diagnosed anxiety disorders. Therefore it is possible that association between anxiety and weight change may differ for those with and without clinical levels of anxiety; however, we were unable to examine this in the current study. Other weaknesses include the exclusion of individuals weighing more than 300 pounds as part of the MESA inclusion criteria; thus, these findings may not be generalizable to morbidly obese individuals.

## 6. Conclusions

This study found no association between trait anxiety scores and weight change over time within a large multiethnic cohort of middle and older aged adults. However, baseline trait anxiety scores were inversely associated with baseline weight, which may indicate that anxiety levels affect weight earlier in life. Future studies should explore the association between anxiety and weight change among individuals during young adulthood.

## Figures and Tables

**Table 1:** Gender-specific anxiety score ranges by Spielberger State-Trait Personality Inventory quartile.

Quartile	Female	Male
Quartile 1	10–13	10–12
Quartile 2	14–17	13–15
Quartile 3	18–20	16–18
Quartile 4	21+	19+

**Table 2:** Baseline characteristics by sex-specific Spielberger State-Trait Personality Inventory quartiles.

Variable	Quartile 1 (*n* = 1,804)	Quartile 2 (*n* = 1,718)	Quartile 3 (*n* = 1,102)	Quartile 4 (*n* = 1,175)
Weight (kg)	79.55 (17.03)	78.76 (17.33)	78.8 (17.16)	78.09 (17.20)
Age	63.07 (9.87)	61.35 (9.90)	60.96 (10.25)	60.79 (10.22)
Percentage of female	54.1	54.2	51.8	48.4
Race/ethnicity (%)*				
White	37.8	40.4	40.5	44.0
Chinese	9.2	12.4	12.6	14.4
Black	32.0	27.1	25.5	19.0
Hispanic	21.0	20.1	21.4	22.6
Income level (%)*				
<$5,000–19,999	19.9	18.4	22.3	27.2
$20,000–49,999	35.0	37.0	37.9	38.5
$50,000–100,000+	45.1	45.6	39.8	34.3
Marital status (%)*				
Married/living as married	62.0	64.4	59.0	59.7
Widowed	14.0	11.6	12.0	10.8
Divorced	13.6	12.7	14.9	13.5
Separated	2.7	3.2	3.3	4.5
Never married	7.0	7.2	9.9	10.6
Prefer not to answer	0.7	1.0	0.9	0.8
Education level (%)*				
No schooling	0.5	0.3	0.6	1.3
Grades 1–8	7.7	6.7	8.7	9.9
Grades 9–11	7.2	5.4	6.6	7.1
High school/GED	16.7	17.6	17.5	21.1
Some college-no degree	16.6	16.1	17.0	15.7
Technical school certificate	7.4	7.1	7.4	6.6
Associate degree	4.8	6.4	4.9	4.3
Bachelor's degree	19.5	18.2	19.0	16.3
Grad./professional degree	19.6	19.9	18.2	17.6

*P < 0.017.

**Table 3:** Results of the mixed effects model examining the relationship between anxiety quartiles and baseline weight (kg).

Variable	Estimate	SE	*P*
Model 1			
Average weight	79.62	0.40	0.000
Average weight change/year	−0.15	0.03	0.000
Baseline weight Q2 versus Q1	−0.95	0.58	0.098
Baseline weight Q3 versus Q1	−0.91	0.65	0.165
Baseline weight Q4 versus Q1	−1.55	0.64	0.016
Model 2			
Average weight	73.54	0.60	0.000
Average weight change/year	−0.15	0.03	0.000
Baseline weight Q2 versus Q1	−1.38	0.54	0.011
Baseline weight Q3 versus Q1	−1.70	0.61	0.005
Baseline weight Q4 versus Q1	−2.71	0.60	0.000
Model 3			
Average weight	81.83	1.13	0.000
Average weight change/year	−0.14	0.04	0.000
Baseline weight Q2 versus Q1	−1.22	0.53	0.022
Baseline weight Q3 versus Q1	−1.61	0.61	0.008
Baseline weight Q4 versus Q1	−2.48	0.60	0.000

Model 1 is unadjusted; Model 2 adjusts for age, sex, race/ethnicity; Model 3 adds the covariates of education level, income level, marital status, and MESA site to Model 2.

**Table 4:** Results of the mixed effects model examining the relationship between anxiety quartiles and weight change (kg/year).

Variable	Estimate	SE	*P*
Model 1			
Average weight	79.62	0.40	0.000
Overall weight change/year	−0.15	0.03	0.000
Weight change Q2 versus Q1	0.04	0.04	0.307
Weight change Q3 versus Q1	0.06	0.04	0.199
Weight change Q4 versus Q1	0.02	0.04	0.727
Model 2			
Average overall weight	73.54	0.60	0.000
Overall weight change/year	−0.15	0.03	0.000
Weight change Q2 versus Q1	0.04	0.04	0.305
Weight change Q3 versus Q1	0.06	0.04	0.199
Weight change Q4 versus Q1	0.02	0.04	0.725
Model 3			
Average weight	81.83	1.13	0.000
Overall weight change/year	−0.14	0.04	0.000
Weight change Q2 versus Q1	−0.02	0.05	0.638
Weight change Q3 versus Q1	−0.02	0.06	0.757
Weight change Q4 versus Q1	0.00	0.06	0.941

Model 1 is unadjusted; Model 2 adjusts for age, sex, race/ethnicity; Model 3 adds the covariates of education level, income level, marital status, and MESA site to Model 2.
